# Physiological response to drought stress in *Camptotheca acuminata* seedlings from two provenances

**DOI:** 10.3389/fpls.2015.00361

**Published:** 2015-05-21

**Authors:** Ye Q. Ying, Li L. Song, Douglass F. Jacobs, Li Mei, Peng Liu, Song H. Jin, Jia S. Wu

**Affiliations:** ^1^The Nurturing Station for the State Key Laboratory of Subtropical Silviculture, School of Forestry and Biotechnology, Zhejiang Agricultural and Forestry University, Lin’an, China; ^2^Department of Forestry and Natural Resources, Hardwood Tree Improvement and Regeneration Center, Purdue University, West Lafayette, IN, USA; ^3^Jiyang College, Zhejiang Agricultural and Forestry University, Lin’an, China

**Keywords:** *Camptotheca acuminata*, provenance, drought stress, physiological response, antioxidant enzyme

## Abstract

Drought stress is a key environmental factor limiting the growth and productivity of plants. The purpose of this study was to investigate the physiological responses of *Camptotheca acuminata* (*C. acuminata*) to different drought stresses and compare the drought tolerance between the provenances Kunming (KM) and Nanchang (NC), which are naturally distributed in different rainfall zones with annual rainfalls of 1000–1100 mm and 1600–1700 mm, respectively. We determined relative water content (RWC), chlorophyll content [Chl(a+b)], net photosynthesis (Pn), gas exchange parameters, relative leakage conductivity (REC), malondialdehyde (MDA) content and superoxide dismutase (SOD) and peroxidase (POD) activities of *C. acuminata* seedlings under both moderate (50% of maximum field capacity) and severe drought stress (30% of maximum field capacity). As the degree of water stress increased, RWC, Chl(a+b) content, Pn, stomatal conductance (Gs), transpiration rate (Tr) and intercellular CO_2_ concentration (Ci) values decreased, but water use efficiency (WUE), REC, MDA content and SOD and POD activities increased in provenances KM and NC. Under moderate and severe drought stress, provenance KM had higher RWC, Chl(a+b), Pn, WUE, SOD, and POD and lower Gs, Tr, Ci, and REC in leaves than provenance NC. The results indicated that provenance KM may maintain stronger drought tolerance via improvements in water-retention capacity, antioxidant enzyme activity, and membrane integrity.

## Introduction

Drought stress is a key environmental factor limiting the growth and productivity of plants (Chaves and Oliveira, 2004). It is particularly true for perennial tree species because they will likely face several water shortages during their lifespan (Perdiguero et al., 2013). Drought conditions are predicted to drastically increase in the next decades, as ongoing climate change is projected to result in more frequent and severe drought periods (IPCC, 2007). Thus, understanding the adaptive responses of tree species that will face these situations is of the utmost interest for plant production.

Drought stress has adverse effects on trees, including leaf water loss, growth inhibition, decreased photosynthetic activity, damaged organelle structures, induced chlorophyll degradation and even accelerated aging processes (Munne-Bosch et al., 2001; Lei et al., 2006; Xiao et al., 2009). Drought also causes an accumulation of reactive oxygen species (ROS) and induces oxidative stress on plant cells (Munne-Bosch et al., 2001; Xiao et al., 2009; Lipiec et al., 2013). Membrane phospholipids are the major targets for ROS, which can mediate membrane damage and lipid peroxidation and increase membrane permeability (Sharma et al., 2012). The effects of drought on plants can be mitigated by a number of strategies. (i) One strategy is related to the presence of protection mechanisms, such as scavenging enzymes and non-enzyme antioxidants, which can reduce the negative consequences of ROS and enhance their drought tolerance (Gill and Tuteja, 2010). Over-production of ROS through chloroplast electron transport chains in plants can be avoided by degrading/retaining chlorophyll and triggering the anti-oxidative scavenging mechanisms to avoid further cell structure damage (This et al., 2000; Farrant et al., 2003). Thus, the control of ROS and the stability of photosynthetic pigments under stress conditions are reported to increase resistance to drought stress (Türkan et al., 2005). (ii) A second strategy is to tolerate plant dehydration by effectively controlling water loss mainly through stomatal closure and improving water use efficiency (WUE; DaMatta et al., 2003). However, acclimation and adaptation of plants to drought stress vary among species and their provenances, which lay the groundwork for plant improvement. For example, WUE in drought-tolerant clones of *Coffea canephora* was better than for drought-sensitive ones (DaMatta et al., 2003). Javed et al. (2013) suggested that different cultivars of safflower exhibited different antioxidant defense in response to drought.

*Camptotheca acuminata* (*C. acuminata*) is a subtropical species indigenous to south China and has become increasingly important due to the presence of camptothecin (CPT) in all plant parts, which has been used in the clinical treatment of ovarian and colorectal cancer (Liu and Adams, 1996). In addition to its medicinal value, the tree has been widely used as a timber species because it possesses strong apical dominance that leads to straight boles and rapid growth. *C. acuminata* has been successfully introduced to several countries (Liu et al., 1999).

*C. acuminata* provenances have natural distributions within the precipitation range of 1000–1900 mm in the south of China (Ying et al., 2012). Provenances Kunming (KM) and Nanchang (NC) are distributed in different rainfall zone with mean annual rainfall of 1000–1100 mm and 1600–1700 mm, respectively. It has been reported that provenances KM and NC had significant differences in growth and biomass production in response to drought stress (Ying et al., 2012). However, geographic variation in physiological characteristics, including photosynthetic ability, membrane injury and antioxidant systems, which may relate to rainfall seasonality in the two provenances’ natural habitats, has yet to be explored. The documented geographic/genetic variation in *C. acuminata* provides an opportunity to investigate the mechanism underlying resistance to drought. We hypothesized that provenance KM, which occurs in the lower rainfall area, has higher tolerance to seasonal drought than provenance NC that is adapted to the higher rainfall area. We therefore compared the drought tolerance between the provenances by analyzing physiological parameters, including chlorophyll content, relative water content (RWC), photosynthesis, lipid peroxidation, and antioxidant enzyme activities to better understand the physiological basis of the survival potential of *C. acuminata* provenances and for selecting provenances for the expansion of *C. acuminata* plantations.

## Materials and Methods

### Plant and Culture

Seeds of *C. acuminata* from two different provenances (Table 1) were obtained and sown in soil for germination in Lin’an of Zhejiang province in March 2009. Seeds of *C. acuminata* were obtained from National Fuxi Forestry Center (Chun’an, Zhejiang Province, China) and sown in soil for germination in Lin’an of Zhejiang province, China. Three-month-old homogenous seedlings were transplanted into plastic pots (13.5-cm inner diameter, 16-cm height, with holes in the bottom) in May 2009, and later transferred to a greenhouse. All the pots were irrigated daily and maintained at 75% field capacity of soil. Seedlings were left to grow for 2 months, and later were exposed to three different levels of drought stress, namely non-water stress (control, watered and maintained at 75% field capacity), moderate water stress (MWS, watered and maintained at 50% field capacity) and severe water stress (SWS, watered and maintained at 30% field capacity), which were randomly assigned to seedlings. Three replicates, each with six seedlings, were used for each treatment. A stratified soil coring procedure was used to determine the maximum field capacity of the soil, and the soil water content was determined by the weighing method. All treatments were kept at 25 ± 2°C and 60% relative humidity (RH) during the day, 22 ± 2°C and 70% RH at night with a 12 h dark/light photoperiod (PPFD, 1200 ± 50 μmol m^–2^ s^–1^). To ensure that the soil water content remained within the gradient, water was replenished at 5:30 pm every day. After 40 days of drought treatment, leaves from six seedlings of each of three replications per treatment were collected and stored at –4°C for further use.

**TABLE 1:** **Information on provenances NC and KM used in the drought stress analysis**.

**Provenance**	**Source**	**Latitude and longitude**	**Average annual temperature (°C)**	**Average annual precipitation (mm)**
NC	Nanchang, Jiangxi	E115°57′, N28°39′	17.5	1600–1700
KM	Kunming, Yunnan	E102°40′, N25°23′	15.0	1000–1100

### Determination of Pigment and RWC

The total chlorophyll content [Chl(a+b)] in the leaves was determined using the 96% ethanol immersion method (Lichtenthaler, 1987). A total of 0.1 g of leaves from 6 seedlings was cut into small pieces (0.2 cm filaments) and extracted with 8 ml of 95% (v/v) alcohol in the dark for 24 h at 25°C until the leaves were blanched. The absorbance of the supernatant was measured at 645 and 663 nm with a Shimadzu UV-2550 spectrophotometer (Kyoto, Japan). The chlorophyll concentrations were calculated by the standard method of Arnon (1949) and expressed as mg/g fresh weight (FW).

The RWC was measured with the dry immersion method (Zhang et al., 2005). After rehydrating for 24 h in the dark (saturated weight, Ws) and oven-drying at 85°C for 24 h to a constant mass (dry weight, Wd), 0.5 g of fresh leaves were weighed immediately (fresh weight, Wf). The RWC was calculated as the following:

RWC(%)=[(Wf−Wd)/(Ws−Wd)]​ ​× 100

### Measurement of Photosynthetic Parameters

After 40 days of drought treatment, leaf photosynthetic parameters were measured with a portable photosynthesis system (LI-6400; LI-COR Inc., Lincoln, NE, USA) with a LED light source (6400-02). Net photosynthesis (Pn), stomatal conductance (Gs), intercellular CO_2_ concentration (Ci) and transpiration rate (Tr) were measured at a light intensity of 1200 μmol m^–2^ s^–1^ PAR, leaf temperature of (25 ± 2)°C and constant CO_2_ of 450 μmol CO_2_ mol^–1^ in the sample chamber provided by buffer volume. Gs to water vapor (mol H_2_O m^–2^ s^–1^) is termed g*_sw_* and obtained from total conductance (g*_tw_*) by removing the contribution from the boundary layer, where K_f_ is a factor based on the estimate K of the fraction of stomatal conductance of one side of the leaf to the other (termed stomatal ratio) and g*_bw_* is the boundary layer conductance to water vapor (mol H_2_O m^–2^ s^–1^) from one side of the leaf.

(1) $$g_{sw}=\frac{1}{\frac{1}{g_{tw}}-\frac{k_{f}}{g_{bw}}}$$

(2) $$k_{f}=\frac{K^{2}+1}{{\left(K+1\right)}^{2}}$$

To avoid the effects of photoinhibition, all measurements were performed under natural conditions between 9:00–11:00 am on sunny days. One seedling from each of three replications per treatment was randomly selected for the measurements.

### Determination of the Relative Electric Conductivity (REC) and Lipid Peroxidation

The REC in leaves was determined using the DDS-11A conductivity meter (Shi et al., 2008). First, 0.2-g disks were briefly rinsed with deionized water and immersed in a test tube with 30 ml of deionized water for 12 h. The electrical conductivity (EC_1_) of the solution was measured with a conductivity meter (Model DJS-1C; Shanghai Analytical Instrument Co., Shanghai, China). The samples were then heated in boiling water for 20 min and cooled to room temperature. The conductivity of the killed tissues (EC_2_) was measured. Membrane permeability was calculated as the ratio of EC_1_/EC_2_.

The extent of lipid peroxidation was estimated by determining the concentration of malondialdehyde (MDA), which was determined by the thiobarbituric acid (TBA) reaction according to the method of Jin et al. (2011a). A total of 2 ml of 0.6% TBA in 10% trichloroacetic acid (TCA) was added to each 2-ml aliquot of the supernatant. The mixtures were heated at 100°C for 15–30 min and then quickly cooled in an ice bath. After centrifugation at 5000 × *g* for 20 min, the absorbance of the supernatant was recorded at 532, 600, and 450 nm. Lipid peroxidation was expressed as μmol g^–1^ with the following formula: 6.45(A_532_ – A_600_) – 0.56A_450_.

### Determination of Superoxide Dismutase (SOD) and Peroxidase (POD) Activities

Leaf tissues (0.5 g) were cut into pieces and ground in 10 ml of 50 mmol phosphate buffer (pH 7.8) containing 1% (w/v) polyvinylpyrrolidone (PVP). The homogenate was centrifuged at 10,000 × *g* for 15 min at 4°C, and the supernatant was then used to determine the SOD and POD activities.

Superoxide Dismutase activity was determined based on the inhibition of nitrobluetetrazolium reduction to blue formazan by superoxide radicals (Giannopolites and Ries, 1977). The reaction mixture (3 ml) consisted of 50 mmol potassium phosphate buffer (pH 7.8) containing 0.3 mol ethylene diaminetetraacetic acid, 39.15 mol methionine, 0.225 molnitrobluetetrazolium, 0.006 mol riboflavin and 0.05 ml of enzyme extract. The reaction was conducted for 15 min at 25°C under 4000 lx. One unit of SOD activity was defined as the amount of enzyme that resulted in a 50% inhibition of nitrobluetetrazolium reduction. The specific SOD activity was expressed as U mg^–1^ protein.

Peroxidase activity was determined with the guaiacol method (Sun et al., 2011). The reaction mixture (3 ml) contained 0.05 ml of enzyme extract, 2.75 ml of 50 mmol phosphate buffer (pH 7.0), 0.1 ml of 1% H_2_O_2_ and 0.1 mL of 4% guaiacol solution. The increase in the absorbance at 470 nm due to guaiacol oxidation was recorded for 2 min. One unit of enzyme activity was defined as the amount of the enzyme causing a change in absorbance of 0.01 per min. The specific POD activity was expressed as U mg^–1^ protein.

### Statistical Analysis

Data analyses were performed with SAS 9.2 (2008) software. All data were tested for normal distribution by Shapiro–Wilk test and for homogeneity of variances by Levene’s test. If the data did not have similar variances, the non-parametric Kruskal–Wallis test was applied. If the results of the Kruskal–Wallis test showed significant differences at the 0.05 significance level, we used the Mann–Whitney test (or the two-sample Kolmogorov–Smirnov test) for multiple comparisons among the different treatments/groups.

## Results

### Changes in the Relative Water Content, Pn and Chlorophyll Content of Provenances NC and KM Under Water Deficit Conditions

**FIGURE 1:**
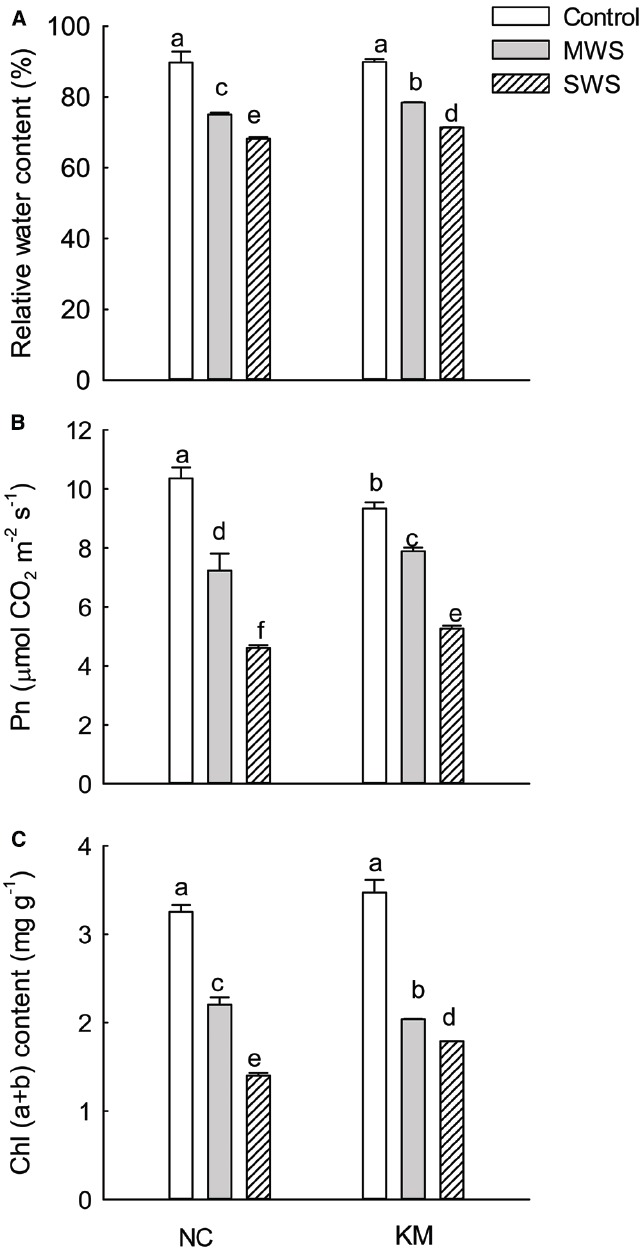
**Changes in RWC (A), Pn (B), and Chl(a + b) (C) of leaves in provenance NC, which grows in a higher rainfall area, and provenance KM, which grows in a lower rainfall area, subject to three different drought treatments (control: watered and maintained at 75% field capacity; MWS: moderate water stress, watered and maintained at 50% field capacity; SWS: severe water stress, watered and maintained at 30% field capacity).** Values are the mean ± SD (*n* = 3). Similar letters indicate non-significant differences among treatments and between provenances at *p* < 0.05 according to Mann–Whitney test.

For the two provenances, seedlings under water stress had lower RWC values than those that were well watered. Compared to the non-stressed seedlings, provenance NC had RWC values that were 16.4 and 24.0% lower under moderate and severe drought stress, respectively, whereas provenance KM had only 12.8% and 20.7% lower RWC under moderate and severe stress, respectively. The RWC in provenance KM seedlings was significantly higher than that in provenance NC under moderate and severe stress (Kruskal–Wallis test, moderate stress: χ^2^ = 3.86, *p* = 0.049; severe stress: χ^2^ = 3.86, *p* = 0.049; Figure 1A).

The Pn in provenance NC was higher than that in provenance KM under non-water stress. As the degree of water stress increased, the Pn in the two provenances decreased rapidly. However, the Pn in provenance KM seedlings was significantly higher than that in provenance NC under moderate and severe stress (Kruskal-Wallis test, moderate stress: χ^2^ = 4.35, *p* = 0.037; severe stress: χ^2^ = 3.86, *p* = 0.049; Figure 1B). Similarly, Chl(a+b) content of the leaves in both NC and KM seedlings significantly decreased with the increasing degree of water stress. Compared to the non-stressed seedlings, the Chl(a+b) content of the leaves in provenance NC decreased by 56.9% under severe drought stress, whereas the Chl(a+b) content in provenance KM decreased by only 48.4% under severe drought stress (Figure 1C). Interestingly, a significant difference in Chl(a+b) was found between provenances NC and KM under severe stress (Kruskal–Wallis test, χ^2^ = 3.86, *p* = 0.049).

### Changes in Gs, Tr, Ci, and WUE of Provenances NC and KM Under Water Deficit Conditions

The change in Gs, Tr and Ci of provenances NC and KM under water deficit conditions is shown in Table 2. The Gs, Tr and Ci values decreased under drought stress. Under severe stress, the Gs, Tr and Ci of provenance NC seedlings decreased by 51.6, 8.5, and 67.9%, respectively. Compared to provenance NC, the decline in Gs, Tr and Ci in provenance KM was higher under severe stress, with 68.6, 15.4, and 75.5%, respectively. Furthermore, provenance KM increased its WUE by 44 and 70.86% under moderate and severe stress, respectively, whereas provenance NC increased its WUE by only 5.48 and 35.25% under moderate and severe stress, respectively (Table 2).

**TABLE 2:** **Changes in Gs, Ci, Tr, and WUE in provenances NC and KM after 40 days of drought treatment**.

**Provenance**	**Treatment**	**Gs mol m^–2^ s^–1^**	**Ci μmol mol^–1^**	**Tr mmol m^–2^ s^–1^**	**WUE**
NC	Control	0.122 ± 0.009a	236.0 ± 8.49a	2.77 ± 0.27a	3.83 ± 0.37b
	MWS	0.080 ± 0.003b	223.5 ± 12.02b	1.84 ± 0.03b	4.04 ± 0.03b
	SWS	0.059 ± 0.004c	216.0 ± 2.83b	0.89 ± 0.09c	5.18 ± 0.58a
KM	Control	0.118 ± 0.003a	230.0 ± 0.9a	2.74 ± 0.26a	3.50 ± 0.31b
	MWS	0.094 ± 0.002b	215.5 ± 2.12b	1.53 ± 0.16b	5.04 ± 0.54a
	SWS	0.037 ± 0.003c	194.5 ± 6.36c	0.67 ± 0.08c	5.98 ± 0.79a

Values are the mean ± SD (n = 3). Different letters indicate a significant difference of one provenance under different treatments at p < 0.05 according to Mann–Whitney test. Drought treatments are control: watered and maintained at 75% field capacity; MWS: moderate water stress, watered and maintained at 50% field capacity; SWS: severe water stress, watered and maintained at 30% field capacity.

### Changes in Membrane Damages and Antioxidant Enzymes of Provenances NC and KM Under Water Deficit Conditions

**FIGURE 2:**
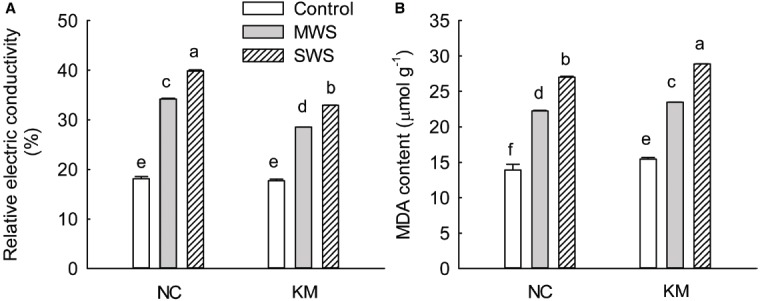
**Changes in REC (A) and the MDA content (B) in provenance NC, which grows in a higher rainfall area, and provenance KM, which grows in a lower rainfall area, subject to three different drought treatments (control: watered and maintained at 75% field capacity; MWS: moderate water stress, watered and maintained at 50% field capacity; SWS: severe water stress, watered and maintained at 30% field capacity).** Values are the mean ± SD (*n* = 3). Similar letters indicate non-significant differences among treatments and between provenances at *p* < 0.05 according to Mann–Whitney test.

As shown in Figure 2A, the REC of the *C. acuminata* seedlings continuously increased with the increasing degree of water stress. Provenances NC and KM increased the REC level by 88.71 and 61.25% under moderate stress and by 119.87 and 86.12% under severe stress, respectively. A significant difference was found in REC between provenances NC and KM under moderate and severe stress (Kruskal–Wallis test, moderate stress: χ^2^ = 3.86, *p* = 0.049; severe stress: χ^2^ = 3.86, *p* = 0.049; Figure 2A). The trend of MDA content was in accordance with the changes in the REC, increasing substantially as drought stress progressed. Provenances NC and KM had increased MDA content by 60.12 and 52.11% under moderate stress and by 94.64 and 86.81% under severe stress, respectively (Kruskal–Wallis test, moderate stress: χ^2^ = 3.86, *p* = 0.049; severe stress: χ^2^ = 3.86, *p* = 0.049; Figure 2B).

**FIGURE 3:**
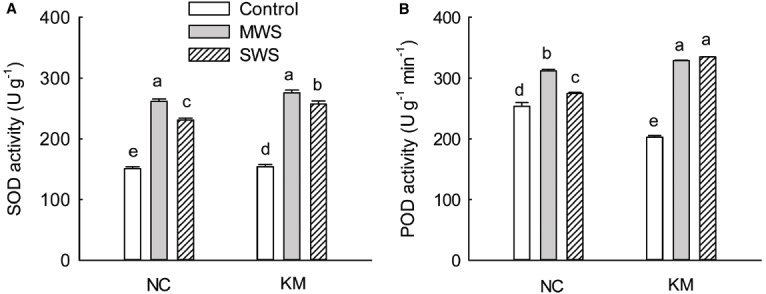
**Changes in the SOD (A) and POD (B) activities in provenance NC, which grows in a higher rainfall area, and provenance KM, which grows in a lower rainfall area, subject to three different drought treatments (control: watered and maintained at 75% field capacity; MWS: moderate water stress, watered and maintained at 50% field capacity; SWS: severe water stress, watered and maintained at 30% field capacity).** Values are the mean ± SD (*n* = 3). Similar letters indicate non-significant differences among treatments and between provenances at *p* < 0.05 according to Mann–Whitney test.

As the degree of water stress increased, the SOD activity of provenances NC and KM increased initially and then declined. Compared with provenance NC, provenance KM had a relatively high level of SOD activity under moderate and severe stress (Figure 3A). Interestingly, the POD activity of provenances NC and KM fluctuated greatly under drought stress. The POD activity of provenance NC was observed to increase by 23.21% under moderate stress and then declined under severe stress, while provenance KM increased POD activity by 62.28% under moderate stress and by 65.24% under severe stress (Figure 3B). At the same time, a significant difference was found in POD between provenances NC and KM under moderate and severe stress (Kruskal–Wallis test, moderate stress: χ^2^ = 3.86, *p* = 0.049; severe stress: χ^2^ = 3.86, *p* = 0.049; Figure 3B).

## Discussion

Tree populations are adapted to prevailing growing conditions through natural selection pressures, which leads to a wide set of physiological and morphological regulatory mechanisms (Zhang et al., 2004). Our results clearly suggest that *C. acuminata* provenance KM, which grows in a lower rainfall area, maintained stronger drought tolerance than provenance NC, which grows in a higher rainfall area, by increasing antioxidant enzyme activity and alleviating membrane permeability and lipid peroxidation.

Relative water content, a key indicator of the degree of cell and tissue hydration, is crucial to optimum physiological functioning and growth processes. Numerous studies have shown that maintenance of a relatively high RWC during drought is indicative of drought tolerance (Jamaux et al., 1997; Altinkut et al., 2001). In this study, RWC decreased in provenances NC and KM with an increasing degree of water stress and seedlings of provenance KM had higher RWC than provenance NC under moderate and severe stress (Figure 1A, *p* < 0.05). Thus, provenance KM, which had a high level of RWC in the leaves, exhibited greater drought stress tolerance as expected given the natural habitat of this provenance. Our findings are consistent with the results in two provenances of *Cakile maritima*, an annual succulent halophyte, experimentally exposed to water deficit (Jdey et al., 2014). Furthermore, in our study, although provenance KM had relatively low Pn under non-water stress, provenance KM maintained a relatively high and stable Pn value compared with provenance NC under severe water stress (Figures 1A,B). This further indicates that provenance KM exhibited a higher level of drought stress tolerance than the NC provenance. Similarly, O’Neill et al. (2006) found that photosynthetic rates of drought tolerant corn hybrids were higher than those of susceptible hybrids. Thus, the ability to maintain key physiological processes, such as photosynthesis, during drought stress varies even among genotypes within a species (Aguilera et al., 1999). This suggests the need to evaluate gas exchange under a broad range of water status conditions to avoid false extrapolation in relative productivity of genotypes.

Stomatal closure, one of the first plant responses to drought, is important in determining overall plant survival strategies (Reynolds-Henne et al., 2010). It is known to be a fast mechanism that reduces water loss and increases CO_2_ diffusion resistance to the mesophyll. In our study, the decrease in Pn was associated with reductions in Gs, Ci, and Tr under moderate and severe water stress (Figure 1; Table 2), which suggests that the main strategy of *C. acuminata* seedlings to endure drought stress is via strict stomatal control. This is in accordance with previous findings (Shen et al., 2014). Interestingly, despite the tight stomatal regulation that we observed under water moderate stress, the decline in Gs, Tr and Ci in provenance KM was much higher than that in provenance NC under severe stress (Table 2), which suggests that provenance KM is better adapted to dry conditions. The high WUE is a direct consequence of the decrease in Gs, which is a typical response previously observed in species that were experimentally subjected to mild drought stress (Medrano et al., 2010). Accordingly, provenance KM was able to maintain high photosynthetic capacity even under severe water stress as a result of increased WUE. This finding was consistent with results for *Eucalyptus microtheca* across its native range in Australia, in which the northwestern provenances with stronger drought tolerance maintained higher leaf water potential than did southeastern provenances (Tuomela, 1997).

Drought stress can accelerate chlorophyll decomposition (Demmig-Adams and Adams, 1996). As expected, we found that the Chl(a+b) content of the leaves in both the NC and KM seedlings significantly decreased with increasing water stress (Figure 2A). Interestingly, provenance KM had a higher level of Chl(a+b) than provenance NC in response to severe drought stress, whereby the Chl(a+b) content of the leaves in KM seedlings under severe drought stress increased by about 28% compared with that of leaves in NC seedlings (Figure 2A). This result further confirms that provenance KM seems to be well adapted to drought stress by protecting chlorophyll from decomposing.

Drought stress negatively affects many aspects of cellular physiology. The major responses to water deficit stress are ROS accumulation, membrane damage and altered antioxidant enzymatic activity, which subsequently lead to the loss of membrane integrity (Li et al., 2011; Jdey et al., 2014). REC is negatively correlated with membrane integrity and reflects the extent of membrane injury (Marangoni et al., 1996). In this study, as the degree of water stress increased, the REC of *C. acuminata* seedlings continuously increased and provenance KM seedlings had a lower level of REC than provenance NC seedlings under moderate and severe stress (Figure 2A), which suggests that drought stress caused membrane damage in *C. acuminata* seedlings and that seedlings of provenance KM experience less tissue damage than those of provenance NC. MDA is a marker of lipid peroxidation in biomembranes (Masia, 2003). Li et al. (2011) reported that contents of MDA and hydrogen peroxide (H_2_O_2_) significantly increased in *Cotinus coggygria* seedlings when experimentally subjected to water deficit (30% of field capacity). Drought stress significantly increased leaf MDA content in both provenances in our study and the increases in MDA content in provenance KM were lower than those in provenance NC (Figure 2B), suggesting less production of ROS in provenance KM subjected to water deficit, thereby leading to relative membrane integrity.

Superoxide dismutase and POD are major antioxidant enzymes that can essentially reduce ROS accumulation, thereby regulating the level of lipid peroxidation (Apel and Hirt, 2004). High POD and SOD enzyme activities may enable the rapid clearance of O_2_^–^ and may catalyze the decomposition of H_2_O_2_ to water and oxygen, thus alleviating the harmful effects of these products (Jin et al., 2011b). Furthermore, some studies have suggested that the activity of antioxidant enzymes, including SOD and POD is correlated with drought tolerance (Türkan et al., 2005). Javed et al. (2013) suggested that different cultivars of safflower exhibited different antioxidant defense in response to drought, who found that THORI-78 can tolerate drought stress well by increasing the activity of catalase and ascorbate POD enzymes whereas variety PI-386174 showed increased activity of the glutathione reductase enzyme under drought. In the present study, as the degree of water stress increased, SOD activity in provenance NC and KM increased initially and then declined (Figure 3A), indicating that SOD enzyme might not be the crucial antioxidant defense during severe drought conditions. Compared with provenance NC, provenance KM increased POD activity by 62.28% under moderate stress and by 65.24% under severe stress. However, the POD activity of provenance NC increased by only 23.21% under moderate stress and then declined under severe stress (Figure 3B). Thus, the high level of drought stress tolerance in provenance KM enhanced POD activity. This is an important mechanism for stress tolerance in *C. acuminata* species and could be relevant for genetic improvement of provenance KM in drought soil conditions.

In conclusion, drought stress decreased RWC, Chl(a+b) content and photosynthetic capacity and increased REC, MDA content and SOD and POD activities in *C. acuminata* provenances KM and NC. Our results showed that provenance KM, which is distributed in lower annual rainfall areas, had higher RWC, Chl(a+b), Pn, WUE, SOD, and POD and lower Gs, Tr, Ci, and REC than provenance NC. Provenance KM may maintain stronger drought tolerance via improvements in water-retention capacity and antioxidant enzyme activity and membrane integrity. The drought tolerance of provenance KM may enhance its potential for climatic adaptations under drier conditions with ongoing climatic change.

### Conflict of Interest Statement

The authors declare that the research was conducted in the absence of any commercial or financial relationships that could be construed as a potential conflict of interest.
